# Safe birth matters: facilitators and barriers to uptake of the WHO safe childbirth checklist tool in a Tanzania Regional Hospital

**DOI:** 10.4314/ahs.v21i1.8S

**Published:** 2021-05

**Authors:** Joycelyn Thomas, Joachim Voss, Edith Tarimo

**Affiliations:** 1 Family Nurse Practitioner-Board Certified, United States Afya Bora Consortium Fellow 2014 – 2015 Franciscan Medical Clinic Federal Way, WA. 98003; 2 Program Director, Doctor of Philosophy in Nursing Program, Frances Payne Bolten School of Nursing, Case Western Reserve University; 3 Muhimbili University of Health and Allied Sciences Dar Es Salaam Afya Bora Working Group Member

**Keywords:** Safe birth matters, WHO safe childbirth checklist tool, Tanzania Regional Hospital

## Abstract

**Background:**

The World Health Organization (WHO) developed the Safe Birth Checklist (SCC) to facilitate best practices in safe birthing practices. The SCC is utilizing existing evidence-based WHO guidelines and recommendations which has combined those into a single and practical bedside tool. The SCC is the first checklist-based intervention to target the prevention of maternal and neonatal deaths.

**Objective:**

The objective of this project was to pilot-test the World Health Organization Safe Childbirth Checklist with Maternity Regional Hospital in, Tanzania.

**Study Design and Methods:**

Retrospective analysis on 35 charts were completed to identify presence or absence of documentation aligned with evidenced based checklist items. Staff training, end user observations and focus group discussions were utilized to elicit feedback about the tool and the process. Descriptive statistics and manual content analysis were used to analyze the rate of uptake and ownership over the checklist. The Checklist is broken down into four sections or time points (that are considered natural pause points in the care of laboring women). The four different pause points are admission, delivery, post-partum, and discharge

**Results:**

We trained 26 participants out of 32 staff how to use the SCC. Delivery time point had the lowest at SCC completion rate at 39% compared to discharge having the highest completion rate at 93%. There was variation in completion rate of the checklist items at each time point. Checklist items at the beginning of each time point were completed between 94% and 100% of the time with the latter checklist list items completed between 29% and 57% of the time

**Conclusion:**

This project was able to identify facilitators and potential barriers to the successful uptake of the Safe Childbirth Checklist in Shinyanga Regional Hospital. Based on these findings, the MOH have opportunities to utilize those findings in the scale-up of the implementation of the checklist and future evaluation activities.

## Introduction and background

Tanzania has shown slow progress towards reducing neonatal and maternal mortality rates by the end of 2015, and has not reached Millennium Development Goals (MDG) 4 and 5 respectively 1. In 2010[JT1] maternal mortality rates were 454/100,000 per live births and neonatal mortality at 26/1000 live births^2^, compared to the global MDG. MDG set expection of maternal mortality at 244/100,000 per live births and neonatal mortality 59/100,000 per live births. Maternal and neonatal death rates globally, are clustered around the time of birth with the majority of deaths occurring within the first 24 hours after delivery 3. Global minimum care standards and best practices for safe childbirth have been well described, including the introduction and worldwide uses of the partograph, a tool to track the progression of labor and alert the clinician if complications require an immediate intervention^4^. A large randomized controlled trial on the use of the partograph that included over 35,000 women, which illustrated reductions in prolonged labors by almost 50% and the proportion of labor requiring augmentation (from 20.7% to 9.1%)^4^. Emergency caesarean sections fell from 9.9% to 8.3%, and intrapartum stillbirths from 0.5% to 0.3%^4^. However, wide adoption of the tool at the bedside by sub-Saharan clinicians is still lacking, resulting in high maternal and infant mortality and morbidity rates^4^. In an effort to improve universal delivery of minimum care standards during childbirth, WHO developed the WHO Safe Childbirth Checklist in consultation with key stakeholders from around the globe. The objective of the Safe Childbirth Checklist-based quality improvement program was to aid Tanzanian health-care workers in reducing the number of adverse events that happen around and after the time of childbirth. The ultimate goal is to train Tanzanian healthcare workers in the use of the SCC and reduce morbidity and mortality of infants and mothers in the first 24 hours surrounding childbirth with the ultimate goal to reduce maternal and newborn morbidity and mortality rates and translate best practices of safe delivery practices in clinical settings.

The primary goals were to pilot-test the World Health Organization Safe Childbirth Checklist with perinatal staff in a single maternity ward in a regional hospital in Tanzaniaand evaluate post implementation facilitators and barriers of the uptake of the SCC.

## Methods

### Stakeholder Sensitization and Champion Engagement

Together with Amref Lake Zone Director, we met with Regional Medical Officer (RMO) for the medical center to introduce the proposed project and gain buy-in. A meeting with the hospital Chief Nursing Officer, an OB/GYN physician, the head nurse for maternity, and the head nurse for the postnatal units followed the initial meeting. Both head nurses facilitated conversation with staff regarding the project. Project champions self-selected and included one OB/GYN medical doctor, the head nurse for maternity, and a postnatal nurse. Participants were introduced to the origin of the Checklist's development and importance of use in maternity care. The participants were able to discuss the use of the checklist during the admission, delivery, postpartum, and discharge and its use during those timepoints.

### Maternity Ward Workflow and Statistics

North Tanzania has a Regional Medical Center that is the single referral center for the region. The hospital has 304-bed capacity serving a catchment area of approximately 1, 567, 038 people (1.5 million). In 2014, there were between 500 and 600 deliveries per month.

The maternity ward is comprised of 4 service areas. Antenatal is a single room with 18 beds, Labor and Delivery is a single room with 5 beds separated by curtains, Postnatal for spontaneous vaginal deliveries was a single room with 19 beds, and Post C-section was a single room with 15 beds total. The ward has a total of 16 registered nurses (RN's) and 16 medical assistants (MA's) and over half of the RN's with additional training in Midwifery. The staff is divided into 3 teams that rotate in an 8-hour shift work throughout the week and the weekends. Daily staffing consists of an RN and MA in Antenatal, an RN and MA in Labor and Delivery, 2 RN's and 1 MA on both postnatal wards per shift. The maternity ward has a total of 3 OB/GYN physicians that rotate between the maternity ward and the gynecology ward.

### Chart Review

A chart review of 35 charts was conducted prior to implementation of the Checklist in March 2015. Eight of the charts were ineligible for review due to discharge prior to delivery. A total of 27 charts were reviewed with 10 charts evaluated for the presence or absence of each item on the 29-item Checklist. The remaining 17 charts were evaluated for presence or absence of Checklist item 2 (starting of the partograph) and Checklist item 7 (administration of oxytocin). Information was collected using an Excel spreadsheet and data was aggregated.

### Checklist Training for Staff

Rogers Diffusion of Innovation Theory helps the researcher to have realistic expectations of the project, which relies on peer-to-peer communication, and is proven a valuable approach when working with very specific populations (5). Buy-in is central to successful adoption according to Rogers. Orientation regarding the pilot project took place over three days with one orientation session per day to sensitize the staff to the project. The training was delivered by an experienced labor and delivery nurse. The training was conducted in English and Swahili and participants were trained on sections of the checklist between 1.5 to 3 hours per training session.

### User Observation

As a first step, to further address any contextual factors prior to use of the tool at bedside, four clinicians using the tool at bedside were observed and notes were collected in a notebook. The observer and the clinical staff did not engage verbally for clarification or questions. Post observation, the tools were collected, and each clinician observed discussed with the observer any facilitators or barriers to utilizing the tool.

### Checklist Implementation

In March 2015, the SCC tool was placed in each patient's chart that was admitted to the obstetric ward. The Checklist contained a total of 29-items (28 items after adaption of tool for the environment). Time point 1 (admission) with 7 items, time point 2 (prior to delivery) with 5 items, time point 3 (immediate post partum) with 9 items, and time point 4 (prior to discharge) with 7 items. At each time point, the nurse, midwife or MA taking care of the patient addressed the items on the checklist. Per the WHO guidelines for use of the tool the checklist should be checked on admission, just prior to pushing or cesarean section, just after birth, and just prior to discharge by the nurses taking care of the patient. The completed checklists were collected and stored in an envelope on the post cesarean section and postnatal wards. To protect confidentiality, there was no identifiable information from the clinician, or the patient collected on the tool. Checklist tools were collected over a total of 5 weeks.

### Focus Groups

A single focus group discussion was conducted to assess the facilitators and barriers to the SCC utilizing purposive sampling methods. Each participant in the focus group discussions signed a consent form. The WHO provided a template for researchers to prepare and conduct focus groups for the SCC implementation. The focus group discussion was comprised of 6 female participants with an average of 5 to 10 years of experience in perinatal. The focus group discussion was conducted in Kiswahili by a Tanzanian physician and lasted about 50 minutes. The facilitator recorded notes from the session. The following questions are examples of what was asked to elicit feedback on the barriers and facilitators of the SCC:

- To what extent have you used the Safe Childbirth Checklist in your facility?
- In your view, what aspects of using the Checklist have been positive and helpful? In what aspects has the Checklist helped you in your work?
- In your view, what aspects of using the Checklist have been more negative or unhelpful? In which specific ways did the Checklist hinder you in your work? In what ways has using the Checklist complicated your work?
- Were there particular items or aspects of the Checklist that posed special difficulty? Why?
- In retrospect, what would you do differently to facilitate use of the Checklist in your facility?
- In retrospect, how do you think use of Checklist could be improved in your facility?

## Results

### Checklist Training and User Observations

A total of 26 participants (out of 32 staff): 15 RN's, and 11 MA's who participated in the training for a total of 3 sessions. During each session light refreshments were provided.. Each of the time points was evaluated and participant's feedback solicited. Feedback from all three sessions were collected as notes and kept in a notebook, summarized and posted on the maternity ward. Staff were able to validate comments from training sessions. The WHO provided a template of the SCC. The tool was translated into Kiswalhili and was slightly modified for the setting based on feedback from training sessions. One of the items was deleted from the Checklist, as it was not applicable to the setting. The tool was observed in the four different service areas (antenatal/triage, LD, surgical theater, post C-section). Many found no problem with the tool and others wish they had ongoing training with the tool.

### Checklist Implementation

Each tool was evaluated for completeness. A tool was defined as complete when at least one checklist item was documented in each of the four sections. During the five-week cycle of collection there was a total of 78 Checklists collected (with varying degrees of completeness) out of approximately 290 births: 46(32.6% completed) the first week, none the second week, 9(66.7% completed) the third week, 10(90.0% completed) the fourth week and 13(30.8% completed) the fifth week. Four tools were excluded as they did not have any sections completed. The total number of tools used for data extraction n=74. Refer to Table 1.

**Table I:** Delivery statistics (maternal and neonatal death are approximate)

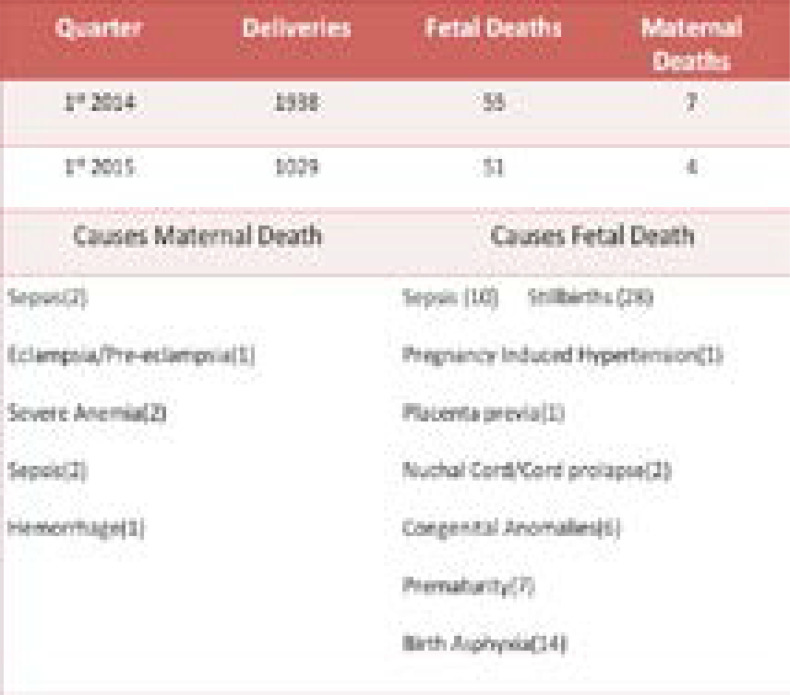

The Checklist is broken down into four sections or time points (that are considered natural pauses in the care of a laboring woman) where the clinician takes a pause to determine what items need to be addressed. The four different pause points are admission, prior to pushing or C-section, within 1 hour post-partum, and prior to discharge with 51, 39, 59, and 74 tools completed respectively, with prior to delivery having the lowest completion rate compared to prior to discharge having he highest completion rate at 93% of the total amounof tools collected. Refer to Table 2. Of the tools completed, between 93 and 100% had a documented signature from a registered nurse with the designation RN.

**Table 2:** Collected checklists

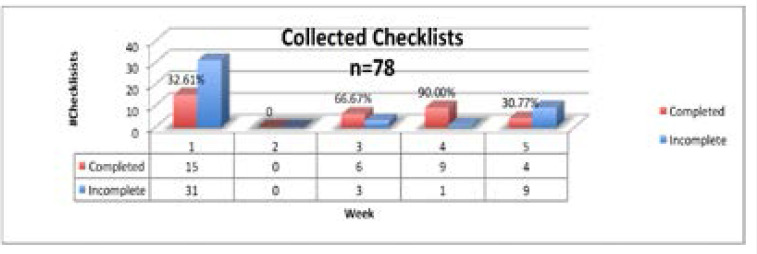

Of the completed time points, admission checklist items 1 though 5 were completed nearly 100% of the time compared to checklist items 6 and 7 which were completed 45% and 29% of the time respectively. Delivery checklist items 1 through 4 were completed between 94% and 100% compared to checklist item 5 with a completion of 35%. Immediate post partum checklist items 1 through 7 completed nearly 100% of the time with checklist items 8 and 9 completed between 38% and 57%. Discharge checklist items 1 through 7 were completed between 95% and 100%.

### Focus Group Discussion

One focus group discussion was conducted in Kiswahili after the last day of collection. The discussion took place in a small office with privacy and enough room to accommodate the 6 participants and facilitator.

### Themes from Focus Group Discussion with Nurses

Themes were extracted using manual content analysis and explored for factors that facilitate the use of the checklist as well as for areas that might hinder use of the checklist in the future. Five key themes relating to facilitators and barriers were identified: Specificity of drug dosing, additional training, simplified language, reminder for essential practices, and adaptation of tool to the environment. Refer to Table 3.

**Table 3:** 

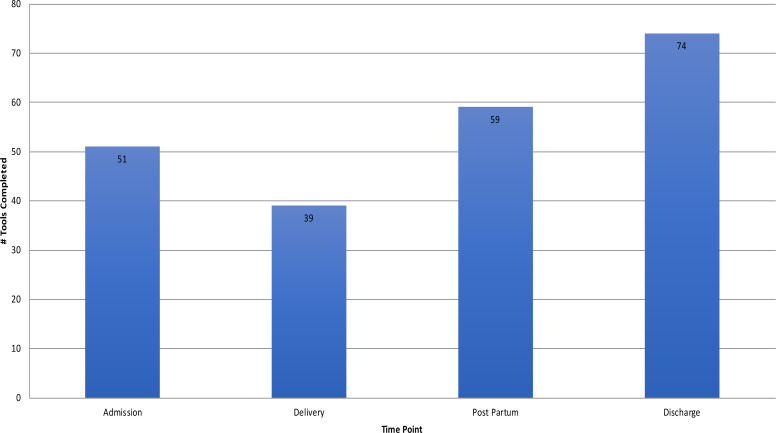

### Facilitators and Barriers to Use of the Checklist

**Figure 1:**
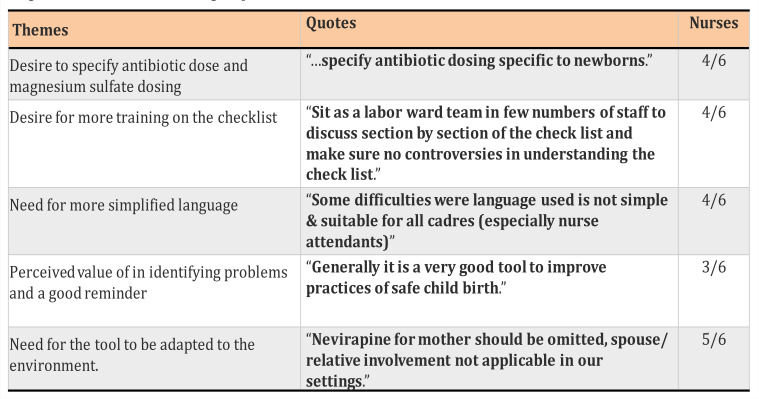
Themes from focus group discussion

Through manual content analysis, four themes were identified as facilitators to the continued use of the tool and five themes were identified as barriers to continued use of the tool (figure 1).

## Discussion

### Implications

The Safe Childbirth Checklist was piloted successfully in a Tanzanian Regional Hospital and facilitators and barriers were identified in the use of the Checklist. Checklist based interventions have been adopted at an increased frequency in health-care to support the clinical care of complex or often neglected tasks that may cause serious human harm. Integration of checklist programs have shown to decrease serious complications in surgical and intensive care settings^5^. Breaking down the process of childbirth into a step-wise process have allowed for the development and implementation of a checklist-based intervention^6^, yet there is little inquiry in the perinatal literature related to checklist programs utilization, facilitators and barriers to use, or outcome measures^7^. Overall, the SCC was well received in Tanzanian Regional Hospital. User observations, data from collected checklists, and themes from focus group discussion validate this point and provide a platform for continued use of the SCC. Findings from the collected checklists demonstrate high utility of the checklist items at each critical pause point suggesting ownership of the tasks by the users and the user-friendly nature of the checklist. However compelling, the last 1 or 2 checklist items at each juncture has dramatic decline in documentation. Inference to the cause of this dramatic decline would be speculative. Rather, multiple factors may contribute to the low utility of these particular checklist-items including but not limited to the need for additional and ongoing training while the checklist is in use, contextual or cultural factors not considered during the project.

Overall, the SCC completion was sporadic. One of the two most prominent omissions is the ongoing assessment of newborn care. Observations of staff validated this finding. Secondly, HIV status is not routinely documented in the mother's chart either by exception charting or in progress notes. Most mothers arrive with an antenatal card that documents their HIV status. The nurses then transfer the HIV status on admission into the admission logbook and once again by the delivery nurses into the delivery logbook. However, there is not a specific place in the flow sheet to document HIV status. An area where the flow sheet documentation measured well against the checklist is with Pitocin documentation of administration. Finally, an area that measured well against the checklist is initiation and use of the partograph. Nearly all charts reviewed showed the utilization of the partograph.

### Strengths

The identified strengths of the project include the high utility of the tool at particular critical junctures, ownership of the tasks outlined in the checklist items as evidenced by the high percentage of clinician signature documentation, leadership buy-in, and reporting by the users and participants in the focus group discussion that the checklist was found to be helpful as a reminder of best practices.

## Limitations

A major limitation to the project is that the implementation was limited to one facility only. Due to the nature of the project and the limited time frame in which to complete the project, a single facility was appropriate. Another limitation was that the focus group discussion was conducted prior to data analysis, which did not allow for identified problem areas to be addressed with questioning during the sessions. However, the participants in the focus group were given the opportunity to respond to an open-ended question asking if there was anything else, they would like to add to the discussion. A lesson learned during this process is the presence of an authority figure during focus group discussion may inhibit the ability of the other participants to freely express their views. Finally, a limitation of the project was the inability to complete more than one cycle of improvement.

## Conclusion, recommendations and future directions

This project demonstrated that uptake of the Safe Childbirth Checklist is possible in Tanzanian Regional Hospital and facilitators and barriers to uptake of the checklist remain similar to other checklist programs. Recommendations for future inquiry should focus on a implementing an intervention study comparing outcomes before and after intervention. Further, scaling up checklist implementation in several facilities using a difference in difference design could generate new knowledge. Future activities should emphasize the importancof such concepts as Super Users or Champions throughout the implementation period. As a WHO Safe Childbirth Checklist Collaboration member, results contributed to the final version of the tool which was released December 4^th^, 2015.
